# Catalytic loop closure governs substrate selectivity in the rhizocticin biosynthetic ligases RhiM and RhiC

**DOI:** 10.1016/j.jbc.2026.113438

**Published:** 2026-08-12

**Authors:** Mizuki Sakai, Ryosuke Masuda, Yu Hirano, Taro Tamada, Akira Nakamura

**Affiliations:** 1 Quantum Life Science Course, Graduate School of Science and Engineering, Chiba University, Inage, Chiba, Japan; 2 Institute for Quantum Life Science, National Institutes for Quantum Science and Technology, Inage, Chiba, Japan; 3 Department of Chemistry, Faculty of Science, Gakushuin University, Toshima, Tokyo, Japan; 4 Center of Quantum Life Science for Structural Therapeutics, Chiba University, Inage, Chiba, Japan; 5 Neutron Industrial Application Promotion Center, Comprehensive Research Organization for Science and Society, Tokai, Ibaraki, Japan; 6 Department of Life Science, Faculty of Science, Gakushuin University, Toshima, Tokyo, Japan

**Keywords:** structural biology, X-ray crystallography, ATPase, enzyme mechanism, substrate selectivity

## Abstract

RhiM and RhiC are ATP-dependent l-amino acid ligases involved in the biosynthesis of the antifungal peptide rhizocticin. RhiM catalyzes the ligation of l-arginine with a phosphonic amino acid, and RhiC subsequently ligates the RhiM product with hydrophobic amino acids. Although biochemical studies have clarified the enzymatic functions of these ligases, the molecular basis of the catalytic mechanisms and substrate selectivity is unknown because there is no structural information for nucleotide- and substrate-bound states or direct structural evidence for the proposed acyl phosphate intermediate. In this study, we performed X-ray crystallographic and biochemical analyses of RhiM and RhiC. Crystal structures were determined for the nucleotide-bound forms of the enzymes as well as the substrate- and intermediate-analog-bound forms of RhiC. The structure of RhiC bound to an intermediate analog provides structural evidence supporting a mechanism where catalysis proceeding *via* an acyl phosphate intermediate. Structural comparisons of the previously reported apo form of RhiM and the multiple reaction-state structures determined here indicate that closure of three catalytic loops is essential for forming the substrate binding pocket and the correct positioning of the substrate and intermediates in the active site. The hydrophilic and hydrophobic pockets formed by these loops are optimally configured to accommodate the RhiM and RhiC substrates. Mutational analyses confirm that residues involved in substrate binding and Mg^2+^ coordination are critical for the ligase activity. These findings show that conformational rearrangements of the catalytic loops govern catalytic reaction and ligand selectivity in these enzymes.

Bioactive peptides produced by microorganisms are crucial in antimicrobial defense (1). Amino acid ligases are ATP-dependent enzymes that catalyze peptide bond formation between amino acids and are involved in the biosynthesis of peptidoglycan and antibiotic peptides. Rhizocticin is a phosphonic acid peptide produced by *Bacillus subtilis* ATCC 6633 (*Bacillus spizizenii* ATCC 6633) and exhibits antifungal activity (2). Rhizocticin consists of a hydrophobic residue, l-Arg, and the non-proteinogenic amino acid (*Z*)-l-2-amino-5-phosphono-3-pentenoic acid (APPA), linked from the N-terminus to the C-terminus (Fig. 1*A*) (3). APPA is structurally similar to the precursor of l-threonine, *O*-phosphono-l-homoserine, and is an inhibitor of fungal l-threonine synthases (1). Because mammals do not possess l-threonine synthase, rhizocticin is expected to be suitable as an oligopeptide antifungal agent.

**Figure 1:**
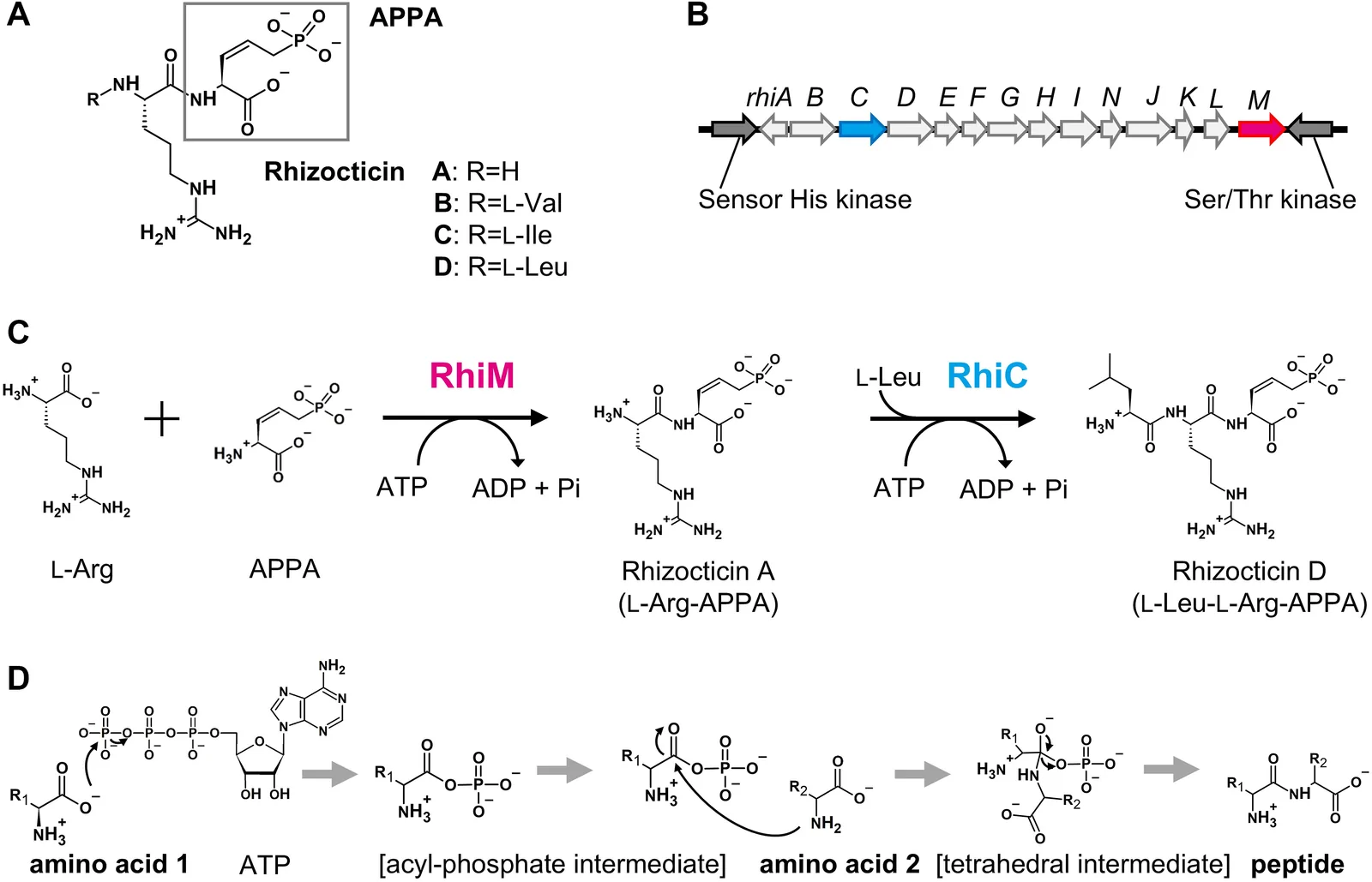
**Rhizocticin biosynthesis.***A*, chemical structures of rhizocticins A*–*D. Their constituent amino acids and the (*Z*)-l-2-amino-5-phosphono-3-pentenoic acid (APPA) moiety are indicated. *B*, *rhi* gene cluster responsible for rhizocticin biosynthesis. The cluster encodes phosphonate biosynthetic enzymes (RhiE, F, G, H), precursor processing enzymes (RhiI, J), and l-amino acid ligases (RhiC, M). *rhiC* is colored *cyan* and *rhiM* is colored magenta. *C*, schematic of ATP-dependent reactions of RhiM and RhiC. RhiM catalyzes ligation between l-Arg and APPA, and RhiC catalyzes ligation between a hydrophobic amino acid (Leu, Ile, or Val) and l-Arg-APPA. *D*, proposed reaction scheme for Lal. The ligation reaction of the first substrate proceeds *via* formation of an acyl phosphate intermediate, in which the ATP γ-phosphate is transferred to the carboxyl group of the first substrate amino acid. Nucleophilic attack on the acyl phosphate intermediate by the second substrate generates a tetrahedral intermediate. After inorganic phosphate is released from the tetrahedral intermediate, the product peptide is formed.

Biosynthesis of rhizocticin involves multiple enzymes encoded in the *rhi* gene cluster (*rhiB*–*rhiM*) in *B. subtilis* ATCC6633 (Fig. 1*B*) (3, 4). This gene cluster is not conserved in *B. subtilis* strain 168, which shares over 90% of its nucleotide sequence with *B. subtilis* ATCC6633 (1). Understanding the molecular mechanisms underlying rhizocticin synthesis by specific enzymes of *B. subtilis* ATCC6633 is important for developing antifungal agents.

RhiM and RhiC, which are encoded in the *rhi* gene cluster, are l-amino acid ligases (Lals) involved in rhizocticin biosynthesis (5). Biochemical analyses suggest that RhiM ligates l-Arg and APPA (5), whereas RhiC ligates l-Arg–APPA and a hydrophobic residue (l-Leu, l-Ile, or l-Val) (6) (Fig. 1*C*). In addition, RhiC has characteristic catalytic activity in Lals that enables the production of peptides longer than a dipeptide (6).

RhiM and RhiC belong to the ATP-grasp superfamily (7), which is characterized by a distinctive fold comprising A-, B-, and C-domains, with the C-domain further divided into C1- and C2-domains (Fig. S1) (8, 9, 10). The ATP-binding motif is conserved in enzymes in this superfamily, and each enzyme has various loop structures (7). The structural flexibility and amino acid sequence diversity confer ligand selectivity and biochemical activity to each enzyme (7).

The three-dimensional structures have been reported for ATP-grasp enzymes, including amino acid ligase (4, 11), acetyl-CoA carboxylase (12), biotin carboxylase (13), and glutathione synthase (14). The X-ray crystal structure of RhiM (also known as RizA) has been determined for its apo form but not for its substrate- and nucleotide-bound forms (4). However, no structural information about RhiC has been reported.

The reaction mechanism of ATP-dependent amino acid ligase has been determined by using d-alanine-d-alanine ligase (Ddl) (14, 15, 16, 17, 18, 19, 20, 21, 22, 23, 24). The proposed mechanism is based on the biochemical analyses and crystal structures of ADP and product-bound forms of Ddl. The γ-phosphate of ATP is transferred to the carboxyl group of one amino acid, generating an acyl phosphate intermediate. This intermediate is then attacked by another amino acid, forming a tetrahedral intermediate. Subsequent release of phosphate results in the formation of the peptide bond between the two amino acids (Fig. 1*D*) (11, 15, 22).

This mechanism differs from that of non-ribosomal peptide synthases (NRPSs), which proceeds *via* an aminoacyl adenylate intermediate (Fig. S2). In the proposed NRPS reaction mechanism, cleavage of the ATP phosphoester bond and formation of the aminoacyl adenylate intermediate are catalyzed during a conformational cycle of the adenylation domain (25, 26).

Thus, Lal and NRPSs generate peptides *via* different pathways, but structural information about the substrate- and nucleotide-bound forms of Lal is limited (4, 11, 27, 28, 29). Furthermore, the structure of the acyl phosphate intermediate has not been determined in amino acid ligases. Therefore, the ligand selectivity and reaction mechanism of amino acid ligases are unclear.

In the present work, to investigate their catalytic mechanisms and ligand recognition, we determined the crystal structures of nucleotide-bound RhiM and of RhiC bound to a nucleotide substrate or nucleotide intermediate analog. Enzyme activity was verified using MS and biochemical assays, and mutational analyses were conducted to identify residues important for the ligase reaction.

## Results

### Crystal structure of ATP-bound RhiM

**Figure 2:**
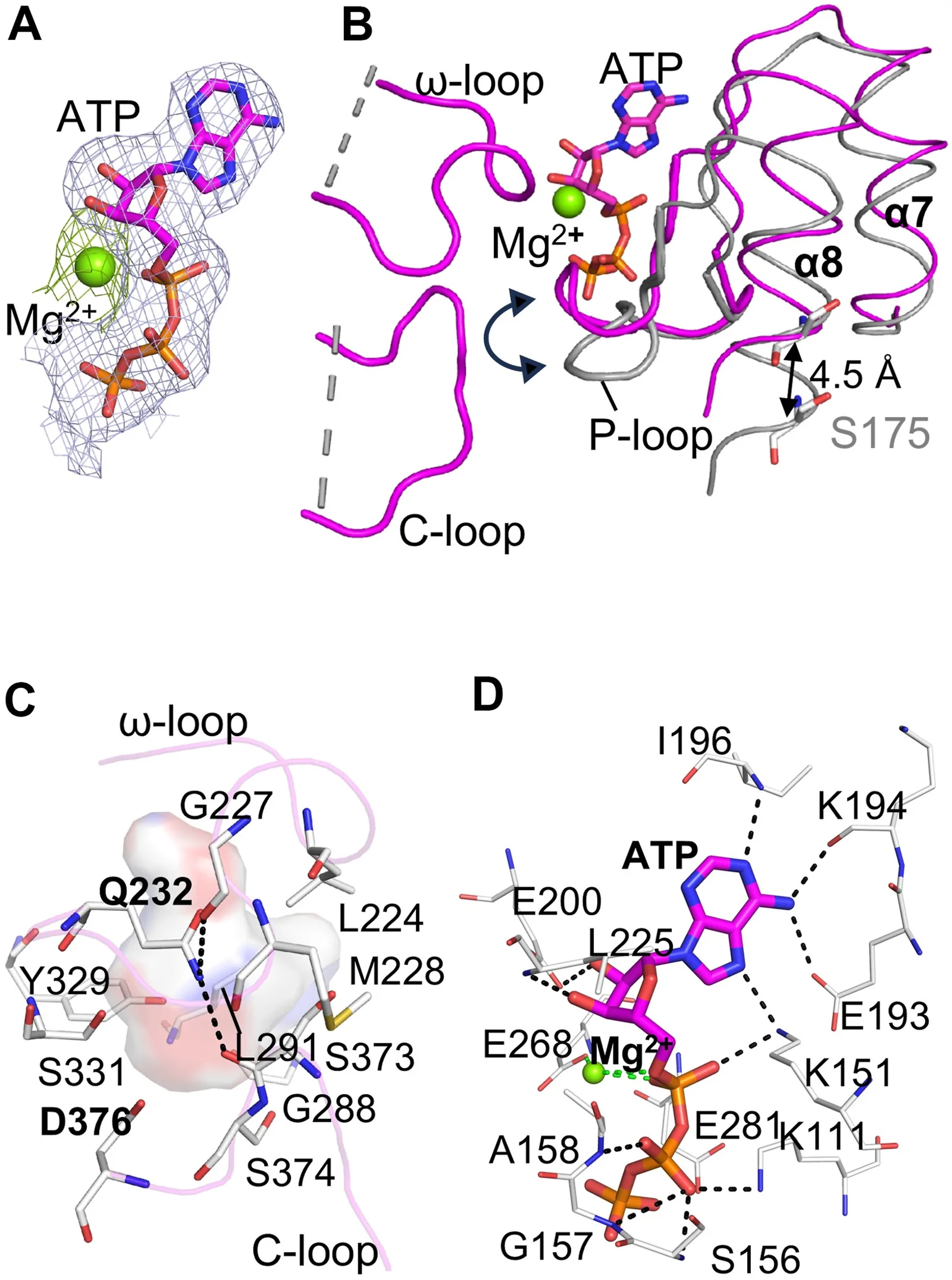
**Structure of ATP–RhiM.***A*, ATP and Mg^2+^ omit maps. The m*F*_o_ − D*F*_c_ electron density maps contoured at 3.0σ are shown as a *light blue mesh* for ATP and a *green mesh* for Mg^2+^. *B*, comparison of the three catalytic loops in ATP bound (*magenta*) and apo (*gray*) forms. The positions of the P-, ω-, and C-loop regions and two α-helices (α7 and α8) are indicated. The ω- and C-loops are disordered in the apo form. Differences in the P-loop and α8 helix are indicated by *arrows*. *C*, substrate-binding pocket. Amino acid residues involved in pocket formation are shown as *sticks*. The pocket surface is *red* (oxygen) and *blue* (nitrogen). *D*, ATP and Mg^2+^ recognition. Residues involved in ATP and Mg^2+^ binding are shown as thin sticks. Dashed lines indicate hydrogen bonds.

Crystals of ATP-bound RhiM (ATP–RhiM) were obtained by co-crystallization of RhiM and ATP. The X-ray crystal structure of the ATP–RhiM complex was determined at 2.45 Å resolution (Table S1). Two RhiM molecules in the asymmetric unit form a dimer, as in the apo form, which is consistent with the gel filtration chromatography results (Fig. S3). ATP and Mg^2+^ binding is confirmed by the m*F*_o_ − D*F*_c_ omit map (Fig. 2*A*). The final model includes residues 1 to 408 of RhiM, ATP, and Mg^2+^, with final *R*_work_ and *R*_free_ factors of 22.4% and 25.6%, respectively (Table S1).

The ATP–RhiM structure reveals the positions of two loop regions at the active site, the ω-loop (T221–I231) and C-terminal loop (V367–D376) (Fig. 2*B*), that were not observed in the apo RhiM structure (4). In the previously reported X-ray structure of a Lal (LdmS) from *Staphylococcus aureus*, the N-terminal loop from the A-domain forms the active site, and the loop is called the N-loop (11). We refer to the C-terminal loop, found in this study, as the C-loop.

To evaluate the conformational changes upon ATP binding, the ATP–RhiM structure determined in this study was superimposed onto the previously reported apo structure (PDB: 4WD3) (Fig. S4). The structures exhibit high structural similarity with a root-mean-square deviation (RMSD) of 0.46 Å for 299 Cα atoms. However, there are differences in two α-helices (α7 and α8) and the phosphate-binding loop (P-loop) in the B-domain (Fig. 2*B*). The Cα atom of S175 at the end of α8 is shifted by 4.5 Å toward the active site in the ATP–RhiM complex. The P-loop between α7 and α8 of apo RhiM is oriented outward from the protein molecule, whereas it is directed toward the active site in ATP–RhiM (Fig. 2*B*).

In the ATP–RhiM complex, the Q232 side chain forms hydrogen bonds with the main chain carbonyl oxygens of G227 and M228 in the ω-loop and with S373 in the C-loop, bridging the two loops (Fig. 2*C*). The resulting slender pocket formed by the ω-loop and C-loop appears optimized for the recognition of l-Arg. Side chains of hydrophilic residues (Q232, S331, S374, and D376) are exposed on the pocket surface, whereas side chains of hydrophobic residues (L224 and L291) are at the pocket entrance (Fig. 2*C*). The residues that form the pocket help to elucidate the substrate recognition mechanism.

The side chains of K151 and E193 and the main chains of K194 and I196 in the B-domain form hydrogen bonds with the ATP adenine ring (Fig. 2*D*). The main-chain carbonyl oxygen of L225 in the ω-loop and the side chain of E200 form hydrogen bonds with the ATP ribose (Fig. 2*D*). The main chains of S156, G157, and A158 in the P-loop form hydrogen bonds with the ATP triphosphate group (Fig. 2*D*). Mg^2+^ is coordinated by the E268 side chain and the ATP α phosphate.

### Overall structures of ligand-bound RhiC

**Figure 3:**
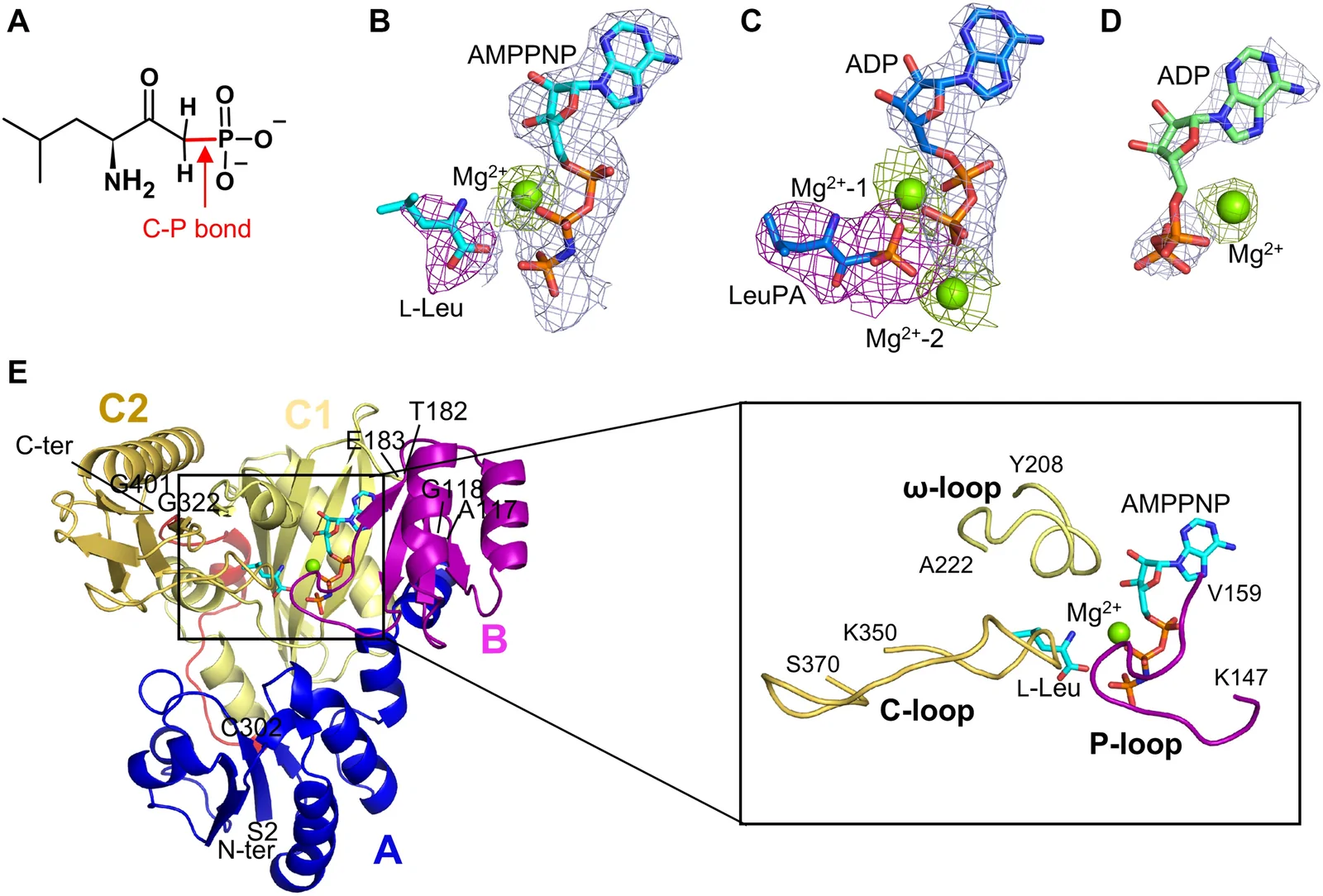
**Structures of ligand-bound RhiC.***A*, chemical structure of the intermediate analog LeuPA. This compound mimics l-Leu acyl phosphate, in which the oxygen atom between the carboxyl and phosphate groups replaced by a carbon atom (methylene group). The non-hydrolysable C–P bond is shown in red. m*F*_o_ − D*F*_c_ omit maps contoured at 3.0σ for (*B*) AMPPNP (*light blue*), l-Leu (*purple*), and Mg^2+^ (*green*); (*C*) ADP (*light blue*), LeuPA (*purple*), and Mg^2+^ (*green*); and (*D*) ADP (*light blue*) and Mg^2+^ (*green*). *E*, (*Left*) Overall structure of the AMPPNP–Leu–RhiC complex. The positions of the domains forming the ATP-grasp fold are indicated. The region (C302–G322) between the C1- and C2-domains is shown in red. The active site is indicated by a square. (*Right*) Close-up view of the three catalytic loops (P-, ω-, and C-loops).

Crystals were obtained by co-crystallization of RhiC with an ATP analog (5′-adenylyl imidodiphosphate; AMPPNP) and l-Leu (AMPPNP–Leu–RhiC), with ADP and an intermediate analog (l-leucyl phosphate analog; LeuPA) (ADP–LeuPA–RhiC), and with ATP and l-Leu (which unexpectedly yielded the ADP-bound form, ADP-RhiC; see below for details). LeuPA ((*S*)-(3-amino-5-methyl-2-oxohexyl) phosphonic acid) is a non-hydrolysable phosphonate analog of the l-leucyl phosphate intermediate, synthesized by replacing the O–P bond between l-Leu and phosphate in l-Leu acyl phosphate with a C–P bond (Fig. 3*A*). The X-ray crystal structures of AMPPNP–Leu–RhiC, ADP–LeuPA–RhiC, and ADP–RhiC were determined at resolutions of 2.90, 3.04, and 2.90 Å, respectively (Table S1). RhiC molecules form a dimer, which is consistent with the gel filtration chromatography results (Fig. S3).

The m*F*_o_ − D*F*_c_ omit map at the active site indicates that AMPPNP, l-Leu, and Mg^2+^ are bound in the AMPPNP–Leu–RhiC complex (Fig. 3*B*), and that ADP, LeuPA, and 2 Mg^2+^ are bound in the ADP–LeuPA–RhiC complex (Fig. 3*C*). Crystals prepared with ATP and l-Leu yielded no electron density for the γ-phosphate in the ADP–RhiC complex, indicating the binding of ADP rather than ATP. Only weak electron density corresponding to l-Leu was detected, making it difficult to model l-Leu or l-leucyl phosphate (Fig. 3*D*).

Figure 3*E* shows the overall structure of the AMPPNP–Leu–RhiC complex. The A-domain consists of residues S2–A117, the B-domain consists of residues G118–T182, and the C-domain consists of residues E183–G401. Residues C302–G322 form a long loop between the C1- and C2-domains. In the AMPPNP–Leu–RhiC complex, a sulfate ion from the crystallization conditions, occupies the positively charged pocket formed by K8 and K13 in the A-domain (Fig. S5 right).

The P-, ω-, and C-loop regions are found in RhiC as well as in RhiM and are formed by residues K147–V159, Y208–A222, and K350–S370, respectively (Fig. 3*E*). In the ADP–RhiC complex, the 2m*F*_o_ − D*F*_c_ map at the active site reveals that the P-, ω-, and C-loop regions are disordered (Fig. S6 right).

Superimposition of these complexes reveals high structural similarity; the RMSD between the AMPPNP–Leu–RhiC and ADP–LeuPA–RhiC complexes is 0.367 Å for 325 Cα atoms, whereas that between the AMPPNP–Leu–RhiC and ADP–RhiC complexes is 0.256 Å for 341 Cα atoms. (Fig. S6 left).

### Ligand recognition of RhiC

**Figure 4:**
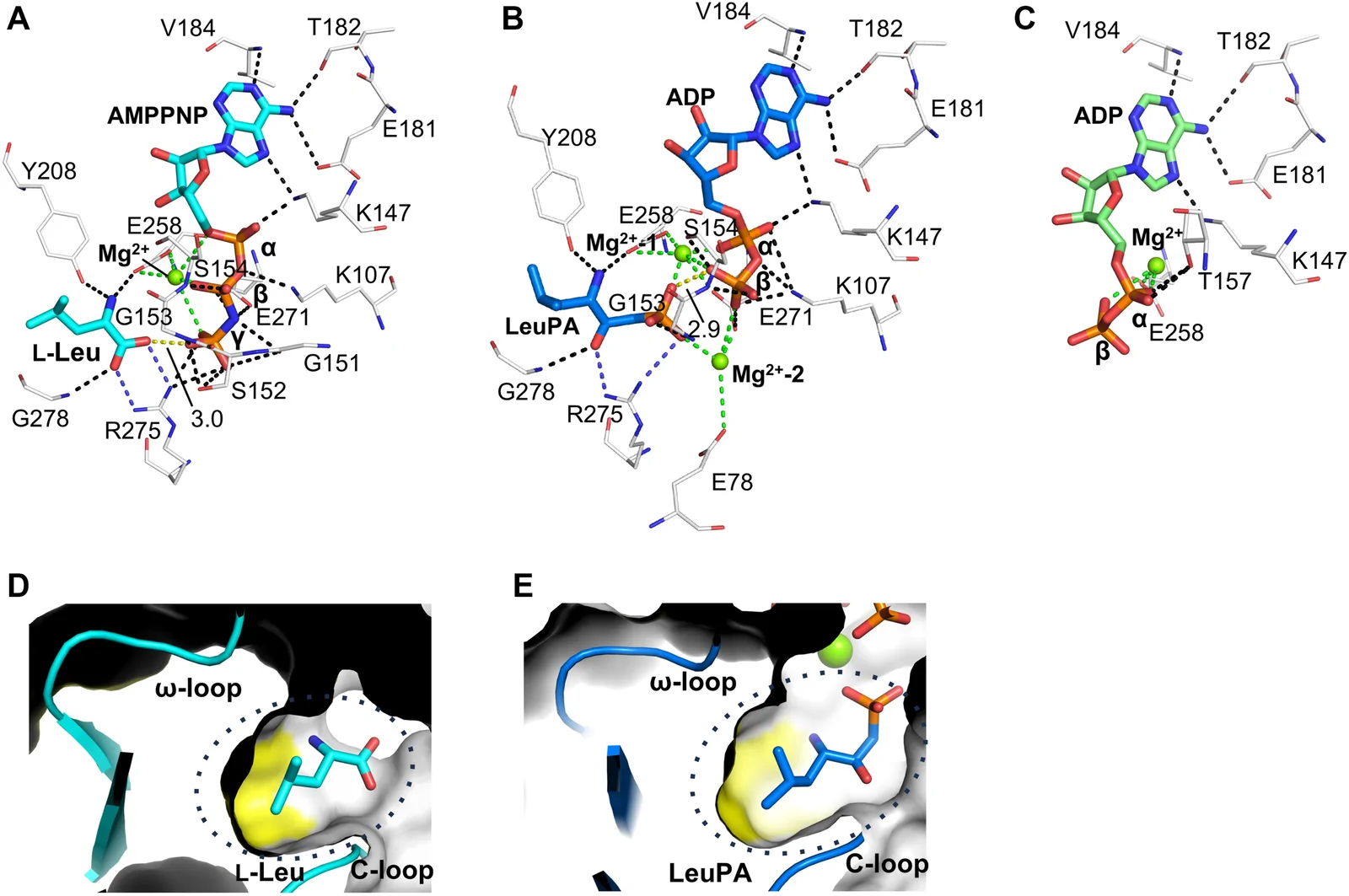
**Interactions at the active site of RhiC.***A*, AMPPNP–Leu–RhiC complex, (*B*) ADP–LeuPA–RhiC complex, and (*C*) ADP–RhiC complex. Residues involved in interactions with the nucleotide, substrate, intermediate, and Mg^2+^ are shown as *sticks*. Hydrogen bonds are indicated by *dashed lines*. The distances between the l-Leu carboxyl group and AMPPNP terminal phosphate and between the LeuPA phosphonate group and ADP β phosphate are indicated in angstroms. *D* and *E*, hydrophobic pockets accommodating the isobutyl groups of (*D*) l-Leu in AMPPNP–Leu–RhiC and (*E*) LeuPA in ADP–LeuPA–RhiC. The ω-loop and C-loop are labeled. l-Leu and LeuPA are shown as *stick* models. Hydrophobic residues on the protein surface are *yellow*.

In the AMPPNP–Leu–RhiC complex, the side chains of K147 and E181 and main chains of T182 and V184 recognize the adenine group of AMPPNP (Fig. 4*A*). The AMPPNP α and β phosphates interact with K107, K147, and S154, and the terminal imido-phosphate interacts with G151, G153, and R275 (Fig. 4*A*). Residues interacting with ADP in the ADP–LeuPA–RhiC and ADP–RhiC complexes are identical to those recognizing the AMPPNP ADP moiety in the AMPPNP–Leu–RhiC complex (Fig. 4, *B* and *C*).

Mg^2+^ in the AMPPNP–Leu–RhiC complex is coordinated by the β and γ phosphates of AMPPNP, and by the side chains of E258 and E271 (Fig. 4*A*). The ADP α and β phosphates coordinate Mg^2+^ (Mg^2+^-1), and the LeuPA phosphoryl group is also involved in the Mg^2+^-1 coordination in the ADP–LeuPA–RhiC complex (Fig. 4*B*). An additional Mg^2+^ (Mg^2+^-2) in the ADP–LeuPA–RhiC complex is coordinated by E78 (Fig. 4*B*). In the ADP–RhiC complex, the position of Mg^2+^ differs from those of Mg^2+^-1 and Mg^2+^-2, and Mg^2+^ is coordinated by E258 (Fig. 4*C*).

The amino group of l-Leu interacts with Y208 and E258, and the carboxyl group of l-Leu interacts with R275 and G278 in the AMPPNP–Leu–RhiC complex (Fig. 4*A*). The carboxyl group of l-Leu is 3.0 Å from the AMPPNP γ phosphate (Fig. 4*A*), implying that a nucleophilic attack of the carboxyl group on the phosphate group occurs in the catalytic reaction (Fig. 1*D*). The isobutyl group of l-Leu is in a hydrophobic pocket (Fig. 4*D*). Residues interacting with the LeuPA amino and carboxyl groups in the ADP–LeuPA–RhiC complex are the same as those interacting with l-Leu in the AMPPNP–Leu–RhiC complex (Fig. 4*B*). The LeuPA phosphoryl group interacts with S152, G153, E258, E271, and Mg^2+^-1 and is 2.9 Å from the ADP β-phosphate (Fig. 4*B*). The LeuPA isobutyl group is in a hydrophobic pocket (Fig. 4*E*), where the l-Leu isobutyl group is bound in the AMPPNP–Leu–RhiC complex (Fig. 4*D*).

### Enzyme assays and mutation analyses of RhiM and RhiC

**Figure 5:**
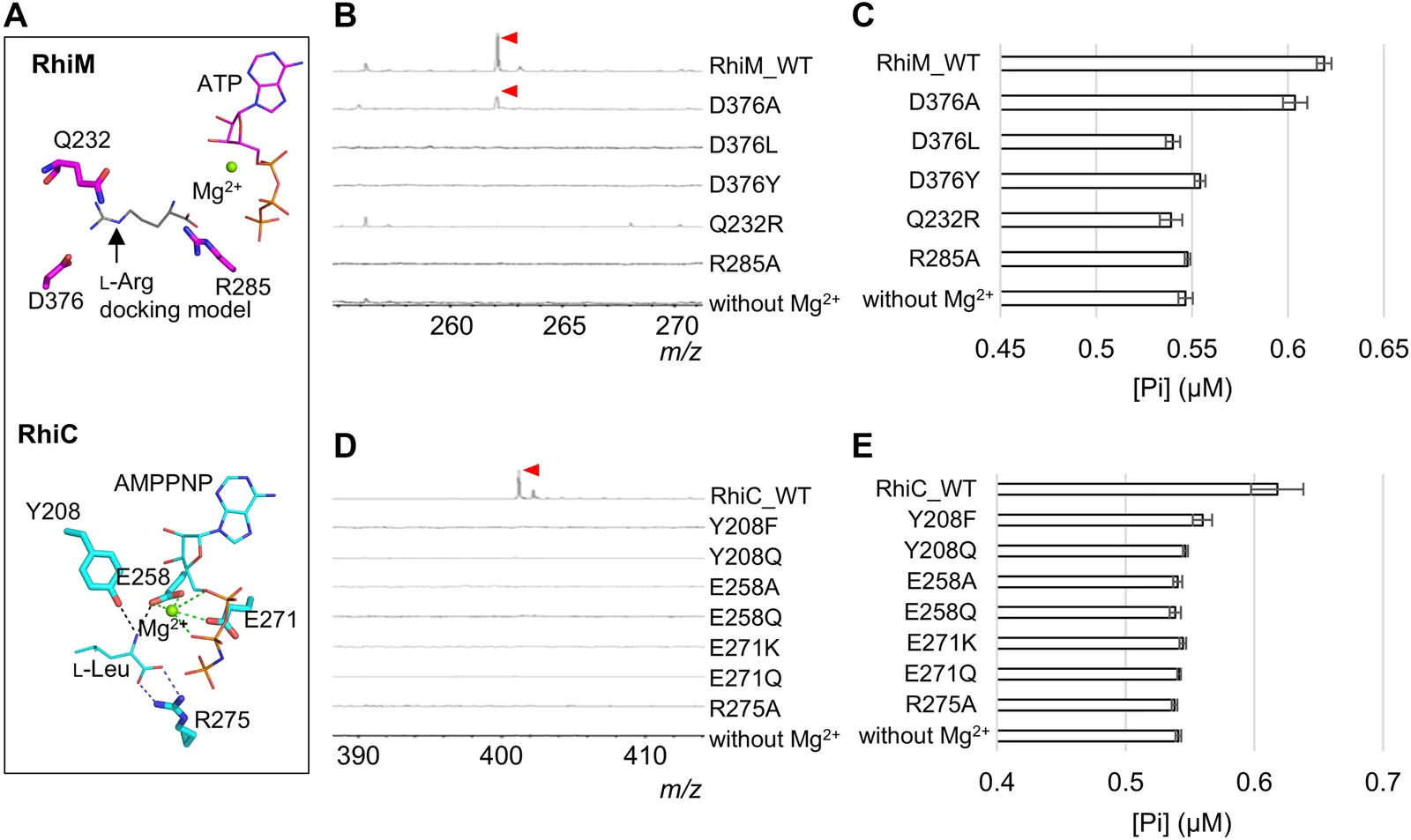
**MS analyses and malachite green assays of RhiC and RhiM.***A*, structural mapping of mutated residues in the active sites. (*Top*) Docking model of RhiM with l-Arg. Mutated residues interacting with l-Arg are shown as sticks. (*Bottom*) Crystal structure of AMPPNP–Leu–RhiC. Mutated residues interacting with l-Leu and Mg^2+^ are shown as sticks. *B*, MALDI-TOF MS spectra of reaction products generated by RhiM. The MS signal corresponding to the product is indicated by a *red arrowhead*. *C*, quantification of inorganic phosphate released during the RhiM reaction using the malachite green assay. Error bars represent standard deviation. Each sample was prepared independently (*n* = 3). Analyses were performed for wild type, mutants (D376A, D376L, D376Y, Q232R, R285A), and wild type without Mg^2+^. *D*, MALDI-TOF MS spectra of reaction products generated by RhiC. The MS signal corresponding to the product is indicated by a *red arrowhead*. *E*, quantification of inorganic phosphate released during the RhiC reaction using the malachite *green* assay. Error bars represent standard deviation. Each sample was prepared independently (*n* = 3). Analyses were performed for wild type, mutants (Y208F, Y208Q, E258A, E258Q, E271K, E271Q, R275A), and wild type without Mg^2+^.

Although we attempted to identify the l-Arg binding site, l-Arg was not detected experimentally in the RhiM structure. Therefore, the binding site was predicted by a docking simulation. The docking models suggest that l-Arg is recognized by R285 and D376 in RhiM (Fig. 5*A*, upper). This Arg residue is conserved between RhiM (R285) and RhiC (R275). Q232 is also likely to be involved in forming the substrate-binding pocket of RhiM (Fig. 2*C*). In RhiC, E258 and E271 are involved in Mg^2+^ coordination, and Y208 and R275 are involved in substrate recognition. To investigate the catalytic roles of these residues, we generated the Q232R, R285A, D376A, D376L and D376Y mutants for RhiM (Fig. 5*A*, upper) and the Y208F, Y208Q, E258A, E258Q, E271K, and R275A mutants for RhiC (Fig. 5*A*, lower).

To evaluate the Lal activity in wild-type and mutant enzymes, we used MS to detect the generated peptide products and the malachite green assay to quantify the inorganic phosphate released during peptide bond formation. In the proposed reaction schemes (Fig. 1, *C* and *D*), the enzymes catalyze ATP-dependent peptide bond formation and concomitantly release the newly formed peptide and inorganic phosphate. Because the natural substrate, APPA, was commercially unavailable and l-Ser functions as a substrate for RhiM, we used l-Arg and l-Ser as substrates for RhiM, and l-Arg and l-Leu as substrates for RhiC as previously reported (5, 6).

MS analysis of the RhiM reaction products revealed that the wild-type and D376A mutant of RhiM produced the l-Arg– l-Ser (R–S) dipeptide, whereas other mutants did not produce a detectable amount of dipeptide (Fig. 5*B*). These results are consistent with the malachite green assay results. The D376A mutant release of inorganic phosphate was comparable to that of the wild-type (Fig. 5*C*). In contrast, the D376L, D376Y, Q232R, and R285A mutants showed inorganic phosphate concentrations similar to that of the wild-type in the absence of Mg^2+^ (Fig. 5*C*). These results indicate that these RhiM mutants have no enzymatic activity.

In RhiC, only the wild type produced peptides (Fig. 5*D*). The tandem mass spectrometry (MS/MS) analysis of the RhiC products indicated that the tripeptide (Leu–Leu–Arg; L–L–R) and tetrapeptide (Leu–Leu–Leu–Arg; L–L–L–R) were produced in addition to the dipeptide (Leu–Arg; L–R) (Figs. S7 and S8). The malachite green assay also demonstrated that all RhiC mutants lost their activity (Fig. 5*E*).

### Structural comparison of RhiM and RhiC

RhiM and RhiC share 56% amino acid sequence homology and 20% identity (Fig. S9). They show high 3D structural similarity, with an RMSD of 2.10 Å calculated over 258 Cα atoms (Fig. S10*A*). The positions of secondary structural elements and the three catalytic loop regions in RhiM and RhiC are highly similar.

The amino acid residues involved in nucleotide recognition and Mg^2+^ (Mg^2+^-1 for ADP–LeuPA–RhiC) coordination are also conserved between the two enzymes (Figs. 2*D*, and 4, *A*, and *B*). The E258 and E271 glutamic acid residues in RhiC, corresponding to E268 and E281 in RhiM, are involved in Mg^2+^ coordination. The loss of catalytic activity caused by the mutations indicates that these two glutamic acid residues are critical for the ligase reaction (Fig. 5, *D* and *E*). Structural comparisons of the ligand-bound forms of RhiC revealed binding variation of Mg^2+^ in the active site (Figs. 4, *A*–*C* and S6). Similar Mg^2+^ rearrangements have been reported in Ddl, in which the Mg^2+^ shift accompanying acyl phosphate intermediate formation is suggested to stabilize the negatively charged environment resulting from phosphate transfer (14, 21, 22, 23). RhiC and RhiM probably follow a similar mechanism, in which the second Mg^2+^ attenuates the electrostatic repulsion of the phosphate groups to stabilize the intermediate complex (Fig. 4*B*).

The carboxyl group of the substrate l-Leu is recognized by the R275 side chain in RhiC (Fig. 4*A*). The corresponding residue, R285, is in a similar position in the active site of the ATP–RhiM complex (Figs. 5*A* and S10*B*). Mutation of the arginine residue caused a loss of activity (Fig. 5, *B*–*D*).

The negatively charged D376 residue in RhiM at the bottom of the substrate-binding pocket has been suggested to be important for recognizing the positively charged side chain of the substrate l-Arg (4). This aspartic acid residue forms a hydrophilic pocket in the active site (Fig. 2*C*). The D376A mutant showed decreased activity compared with wild-type RhiM (Fig. 5, *B* and *C*). These results suggest that although D376 is not essential for RhiM catalysis, it is important in the substrate l-Arg recognition. Mutation of Q232, which connects the ω- and C-loops to form the pocket surrounded by hydrophilic residues in RhiM (Fig. 2*C*), also caused a loss of activity (Fig. 5, *B* and *C*).

In contrast to RhiM, RhiC recognizes hydrophobic residues (l-Leu, l-Ile, and l-Val) as substrates. The isobutyl group of l-Leu is accommodated in the hydrophobic pocket formed by the ω- and C-loops (Fig. 4*D*). The size of this pocket is optimal for the binding of these hydrophobic residues, and the pocket architecture is consistent with the high selectivity for hydrophobic residues with small side chains, as observed in the biochemical analysis (6).

## Discussion

At the second substrate-binding site, the space on the A-domain side of RhiC can accommodate l-Arg–APPA, the product of RhiM (Fig. S5 right). Within this pocket, a bound sulfate ion suggests that the phosphoryl group of APPA interacts at this position. This site is also thought to bind the di- and tri-peptide substrates required for forming the tri- and tetra-peptides identified as RhiC reaction products in this study (Fig. S8). Notably, the conformations of the A-domains are highly conserved among AMPPNP–Leu–RhiC and ADP–LeuPA–RhiC, indicating that this binding pocket has a rigid and pre-formed architecture (Fig. S6*A*). Consistent with these structural features, *in vitro* activity assays of RhiC revealed that its selectivity for hydrophobic amino acids at the first substrate position dictates the reaction sequence. This suggests that tripeptides such as Val–Leu–Arg and Leu–Leu–Arg are synthesized by the addition of Val or Leu to the N-terminus of a pre-formed Leu–Arg dipeptide (Fig. S8). This proposed sequence was experimentally validated by MS/MS analysis of the RhiC reaction products obtained using l-Val or l-Leu alongside the Leu–Arg dipeptide as substrates.

The structure determined in this study represents each of the following states. In the ATP–RhiM structure, ATP is bound whereas the substrate is unbound in a closed conformation poised for substrate binding. The substrate binding pocket is formed by coordinated movements of three loop regions (Fig. 2*B*). AMPPNP–Leu–RhiC mimics a reaction-ready state in the first step of the Lal reaction, in which phosphate transfer from ATP to the first substrate amino acid can proceed. In this structure, the γ phosphate of AMPPNP is close to the carboxyl group of the substrate owing to the closed conformation of the three catalytic loops (Figs. 3*E* and 4*A*). The ADP–LeuPA–RhiC structure corresponds to the post-phosphate transfer state of the first substrate (Fig. 4*B*). Structural evidence from the complex with the acyl phosphate intermediate analog confirms that the intermediate is stabilized by the closed conformation of the three catalytic loops (Fig. S6*B*). In the ADP–RhiC complex, the reaction is complete and ATP hydrolysis has occurred before structural determination. The substrate l-Leu is barely bound, and the electron density corresponding to ADP is ambiguous (Fig. 3*D*). In addition, the three catalytic loops are disordered in the ADP–RhiC complex (Fig. S6*B*).

Although we did not determine the complex structure of a tetrahedral intermediate analog in this study, it has been reported for other Lals. BacD, a Lal from *B. subtilis* 168, catalyzes a ligase reaction using l-Ala as its first substrate (29). Amino acid residues involved in Mg^2+^ coordination and recognition of the substrate carboxyl group are conserved among RhiM, RhiC, and BacD. The crystal structure of BacD with a tetrahedral intermediate analog (PDB: 3VMM) revealed that Mg^2+^ bridges the phosphate of the tetrahedral intermediate analog and the phosphate of ADP (Fig. S11) (29). This Mg^2+^-mediated bridge is similar to the Mg^2+^-1 coordination observed in RhiC bound to the acyl phosphate intermediate analog (Fig. 4*B*). These structural similarities indicate that the Mg^2+^-mediated bridge is essential for the proper positioning of intermediates in the active site.

**Figure 6:**
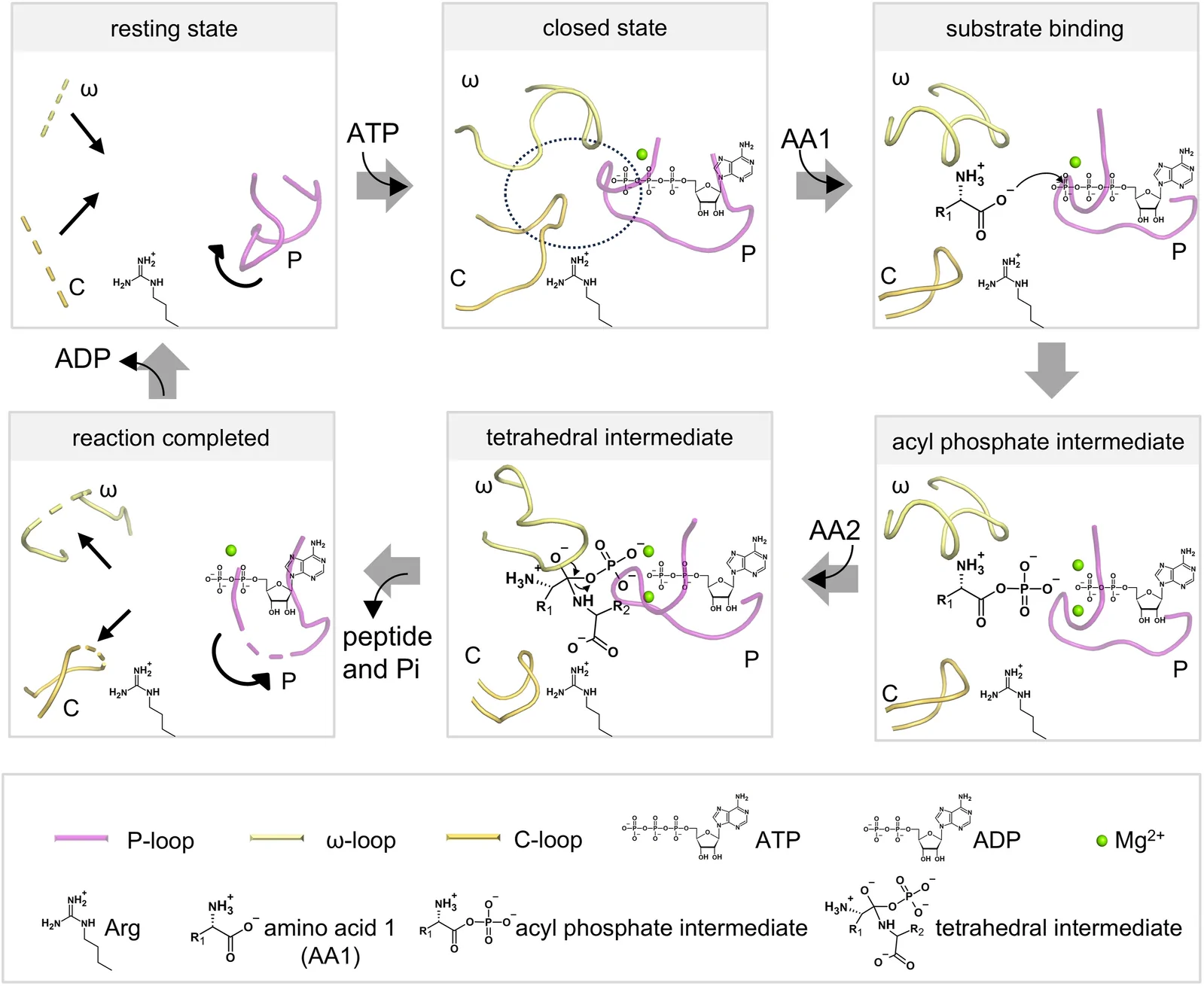
**Proposed reaction mechanism of RhiM and RhiC.** (*Top left*) Resting state before the reaction begins and after it ends. Because the substrate amino acid and ATP are not bound, the ω-loop and C-loop are flexible. (*Top center*) ATP is bound and the substrate amino acid is not yet bound, indicating that the protein is ready to accept the substrate amino acid. When the loops assemble, the active site adopts a closed conformation, forming a pocket. The conserved arginine residue (R285 in RhiM and R275 in RhiC) is in the active site to position the substrate amino acid properly. (*Top right*) The substrate amino acid is bound to ATP, indicating that the phosphate transfer reaction from ATP to the first substrate amino acid is ready to proceed, with the active site adopting a closed conformation. (*Bottom right*) Phosphorylation reaction of the first substrate amino acid. The P-loop, ω-loop, and C-loop are closed, and the acyl phosphate intermediate is held in the pocket by bonds mediated by the P-loop, the arginine residue, and Mg^2+^. (*Bottom center*) Tetrahedral intermediate state. Similar to the acyl phosphate intermediate, the tetrahedral intermediate binds to the enzyme *via* Mg^2+^ through a similar mechanism. (*Bottom left*) The reaction is complete, with the substrate and products released and the active site opened. ATP has been hydrolyzed, the substrate amino acid is not bound, and the electron density of ADP is unclear. All loop structures are disordered.

Based on structural and biochemical analyses, we propose a ligation reaction mechanism involving RhiM and RhiC (Figure 6). ATP binding to the active site of the apo enzyme induces the assembly of the three catalytic loops, adopting a closed conformation and forming the substrate binding pocket. The first substrate is positioned through interactions with the specific arginine residue (R285 in RhiM and R275 in RhiC) and Mg^2+^ and with the substrate-binding pocket, allowing transfer of the ATP γ-phosphate group to the carboxyl group. The resulting acyl phosphate intermediate is then attacked by the amino group of the second substrate, which is bound at the side of the A-domain. After the tetrahedral intermediate is formed, a peptide bond is formed upon the release of the phosphate group from the intermediate. Conformational changes associated with the nucleotide and intermediate states promote the disordering of the catalytic loops, which facilitates the dissociation of ADP and the product.

This study verifies the presence of the acyl phosphate intermediate in the Lal reaction and proposes reaction steps accompanied by loop movements and substrate selectivity arising from the structural differences in the substrate binding pocket. The rational design of substrate selectivity modulation may contribute to commercial applications, including enzyme engineering for synthesizing novel, unnatural, and bioactive peptides.

## Experimental procedures

### Cloning and expression of RhiM and RhiC protein

The *rhiM* and *rhiC* genes were independently cloned from the *B. subtilis* ATCC6633 (*B. spizizenii* ATCC 6633) genome (NBRC 3134) using a primer set (Table S2) and inserted into the pET-21a vector (Merck Millipore) with SLiCE system (30, 31).

*Escherichia coli* BL21(DE3) cells were transformed using the constructed plasmid and cultured in 10 ml of LB liquid medium containing 50 μg/ml ampicillin (LB/Amp) at 37 °C for 16 h (pre-culture). The 4 ml of pre-culture was inoculated into 400 ml of LB/Amp medium, and the cells were grown until the OD_600_ reached 0.5 to 0.7. The culture was placed on ice for about 30 min, and then IPTG (isopropyl β-D-1-thiogalactopyranoside) was added to a final concentration of 0.3 mM. After 18 h culture at 18 °C, the cells were harvested at 5000 rpm and stored at −80 °C.

### Protein purification

The cells were suspended in 35 ml of wash buffer (50 mM Tris-HCl pH 8.0, 20 mM imidazole pH 8.0, 100 mM NaCl, 1 mM dithiothreitol [DTT]) and disrupted by sonication using SONIFIRE 250D (BRANSON). The insoluble fraction was removed by centrifugation at 15,000 rpm for 30 min at 4 °C, and then the supernatant was applied onto 1 ml of Ni-NTA Superflow equilibrated with 20 ml of wash buffer for the initial purification. The column was washed with wash buffer, and the target His-tagged proteins were eluted with seven column volumes of elution buffer (50 mM Tris-HCl pH 8.0, 200 mM imidazole pH 8.0, 100 mM NaCl, 1 mM DTT).

The eluate was applied onto a Superdex200 10/300 GL column equilibrated with gel filtration buffer (50 mM Tris-HCl pH 8.0, 100 mM NaCl, 1 mM DTT) using ÄKTA explorer (GE Healthcare). The purity and homogeneity of the proteins were confirmed by their single, symmetric peaks in gel filtration chromatography (Fig. S3). The purified proteins were concentrated to 14.3 mg/ml (RhiM) and 31.0 mg/ml (RhiC) in the gel filtration buffer and stored at −80 °C until use.

### Chemical synthesis of acyl phosphate intermediate analog

The acyl phosphate intermediate analog was chemically synthesized in two steps: the synthesis of *N*-Boc-Leu-CH_2_P(O) (OMe)_2_, followed by the synthesis of H-Leu-CH_2_P(O) (OH)_2_.

Tetrahydrofuran (THF) and Et_2_O (anhydrous) were purchased from Kanto Chemical and passed through a Kayama Oxygen solvent purification system prior to use. CH_2_Cl_2_ (anhydrous) was purchased from Kanto Chemical and used as received. CDCl_3_ was passed through a small column of neutral alumina prior to use. Bromotrimethylsilane was purchased from Sigma-Aldrich and transferred to J-Young tubes under an argon atmosphere. Other chemicals were purchased from commercial sources and used as received. Silica gel column chromatography was performed using Kanto silica gel N40 to 50. ^1^H NMR spectra were recorded on a JEOL ECZ-400 and the chemical shifts of ^1^H are referenced to the residual proton signal of CDCl_3_ (δ 7.26) and D_2_O (δ 4.65). ^13^C {^1^H} NMR spectra were recorded on a JEOL ECZ-400, and the chemical shifts are given relative to CDCl_3_ (δ 77.00). ^31^P{^1^H} NMR spectra were recorded on a JEOL ECZ-400, and the chemical shifts of ^31^P are referenced to H_3_PO_4_ (δ 0.0) as an external standard. All spectra were assigned with the aid of DEPT, COSY, HMQC, and HMBC NMR experiments. Melting points (m.p.) were measured using an OptiMelt instrument (Stanford Research Systems).

The first reaction was performed according to a reported procedure with slight modification (32). Briefly, a 500 ml three-necked flask was flame-dried *in vacuo*. After it was evacuated and backfilled with nitrogen, THF (150 ml) and dimethyl methylphosphonate (9.88 ml, 92.4 mmol) were added at −78 °C. To the solution, *n*-BuLi in hexane (1.6 M, 57.8 ml, 92.4 mmol) was added dropwise at −78 °C for 30 min, which afforded a yellow solution. The resulting mixture was stirred at the same temperature for 30 min. A THF (100 ml) solution of *N*-Boc-Leu-OMe (6.47 g, 26.4 mmol) in 100 ml of THF, which was prepared in a separate 100 ml two-necked flask, was added dropwise *via* a cannula at −78 °C for 30 min. The resulting pale-yellow solution was stirred at −78 °C for 50 min and then at −30 °C for an additional 2 h before AcOH (2.4 ml) was added. The resulting mixture warmed to room temperature and poured into sat. aq. NaHCO_3_ (100 ml). The two layers were separated, and the aqueous layer was extracted with AcOEt (3 × 40 ml). The combined organic layer was washed with brine, dried over Na_2_SO_4_, and filtered. The filtrate was concentrated *in vacuo*, and the crude product was purified by flash column chromatography on silica gel (eluted with AcOEt only) to give the title compound as a colorless oil. Yield 6.17 g (18.3 mmol, 69%). 1: colorless oil. ^1^H NMR (400 MHz, CDCl_3_): δ 0.93 to 0.98 (m, 6H, CH(CH_3_)_2_), 1.38 to 1.48 (m, 1H, CH(CH_3_)_2_), 1.45 (s, 9H, Boc), 1.62 to 1.77 (m, 2H, CH_2_CH(CH_3_)_2_), 3.09 (dd, ^2^*J*_H–P_ = 22.0 Hz, ^2^*J*_H–H_ = 14.2 Hz, 1H, CH_2_P(O)), 3.32 (dd, ^2^*J*_H–P_ = 22.4 Hz, ^2^*J*_H–H_ = 14.2 Hz, 1H, CH_2_P(O)), 3.79 (dd, *J* = 11.4, 3.7 Hz, 6H, P(O) (OCH_3_)_2_), 4.28 to 4.39 (br m, 1H, CH_2_CH(CH_3_)_2_), 5.17 (br, 1H, NH); ^13^C{^1^H} NMR (100 MHz, CDCl_3_): δ 21.4, 23.2, 24.7, 28.2, 37.2, 38.5, 39.7, 53.0 (^1^*J*_C–P_ = 5.8 Hz), 58.7, 79.9, 155.5, 202.4 (^2^*J*_C–P_ = 6.7 Hz); ^31^P{^1^H} NMR (162 MHz, CDCl_3_): 23.

The second reaction was performed according to a reported procedure with slight modification (32). A 50 ml two-necked flask was flame-dried *in vacuo*. After it was evacuated and backfilled with nitrogen, *N*-Boc-Leu-CH_2_P(O) (OMe)_2_ (1; 2.29 g, 6.80 mmol), CH_2_Cl_2_ (14 ml), and bromotrimethylsilane (7.06 ml, 54.4 mmol) were added at 0 °C. The resulting mixture was stirred at room temperature for 19 h and then concentrated *in vacuo* before adding MeOH (40 ml). The resulting colorless solution was stirred at room temperature for 2 h and concentrated *in vacuo* again. The residue was dissolved in MeOH (4 ml) and reprecipitated with Et_2_O to give the title compound as colorless crystals. Yield 1.55 g (5.33 mmol as HBr salt, 78%). 2: colorless crystals; m.p. 136.9 °C (decomp.). ^1^H NMR (400 MHz, D_2_O): δ 0.81 (d, *J* = 6.9 Hz, 3H), 0.83 (d, *J* = 6.4 Hz, 3H), 1.43 to 1.61 (m, 1H), 1.53 to 1.65 (m, 1H), 1.70 to 1.77 (m, 1H), 3.16 (s, 2H), 4.22 (dd, *J* = 10.1, 3.7 Hz); ^31^P{^1^H} NMR (162 MHz, D_2_O): 12.

### Crystallization, data collection, structure determination, and refinement

ATP and l-Arg were added to the RhiM protein solution to a final concentration of 12.5 mM each. RhiC protein solutions were mixed with AMPPNP (12.5 mM), l-Leu (12.5 mM), and l-Arg (12.5 mM) (AMPPNP–Leu–RhiC), with ADP (12.5 mM) and LeuPA (12.5 mM) (ADP–LeuPA–RhiC), and with ATP (12.5 mM), l-Leu (12.5 mM), and l-Arg (12.5 mM) (ADP–RhiC), as indicated. Crystallization of RhiM and RhiC was performed using the sitting drop vapor diffusion method at 20 °C. Crystallization drops were prepared by mixing equal volumes (1.0 μl + 1.0 μl) of the protein–ligand complex solution and the reservoir solution.

Crystals of the ATP–RhiM complex were grown in a reservoir solution containing 60 mM sodium citrate tribasic dihydrate (pH 5.0) and 10.8% (w/v) PEG 20,000. Prior to data collection, the crystals were soaked for 3 s in a cryoprotectant solution consisting of the reservoir solution supplemented with 20% (v/v) ethylene glycol and flash-cooled in liquid nitrogen.

Crystals of the AMPPNP–Leu–RhiC complex were obtained using a reservoir solution containing 50 mM MOPS, 12 mM NaOH, 28% (v/v) 15/4 pentaerythritol ethoxylate (EO/OH), 50 mM ammonium sulfate (pH 6.7), 12.5 mM AMPPNP, 12.5 mM l-Leu, and 12.5 mM l-Arg. Before flash-cooling, the crystals were soaked for 40 s in a solution containing 28% (v/v) 15/4 EO/OH, 12.5 mM AMPPNP, 37.5 mM MgCl_2_, 37.5 mM l-Leu, and 12.5 mM l-Arg.

Crystals of the ADP–LeuPA–RhiC complex were obtained using a reservoir solution containing 50 mM MOPS, 12 mM NaOH, 28% (v/v) 15/4 EO/OH, 50 mM ammonium sulfate (pH 6.7), 12.5 mM ADP, and 12.5 mM l-LeuPA. Before flash-cooling, the crystals were soaked for 40 s in a solution containing 28% (v/v) 15/4 EO/OH, 12.5 mM ADP, 37.5 mM MgCl_2_, and 12.5 mM l-LeuPA.

Crystals of the ADP–RhiC complex were grown in a reservoir solution consisting of 50 mM MOPS, 10 mM NaOH, 28% (v/v) 15/4 EO/OH, 50 mM ammonium sulfate (pH 6.6), 12.5 mM ATP, 12.5 mM l-Leu, and 12.5 mM l-Arg and were directly flash-cooled in liquid nitrogen without the use of cryoprotectants or ligand soaking.

X-ray diffraction data were collected at the BL-1A, 5A, and 17A beamlines of the Photon Factory. Diffraction intensities were indexed and integrated using *XDS* (33) and merged and scaled using *AIMLESS* in the CCP4 suite (34). The structure of the ATP–RhiM complex was determined by the molecular replacement method using the RizA (apo–RhiM) structure (PDB ID: 4WD3) as a search model with *MOLREP* (35). For the structure determination of the RhiC complexes, the AlphaFold-predicted structure was used as the search model with *Phaser* (36). Manual model building and structural refinement were carried out using *Coot* (37) and *Phenix* (38), respectively. Figures were prepared using *PyMOL*. Substrate docking models for RhiM were generated using *AutoDock Vina* (39).

### Mass spectrometry

RhiC (0.4 μM) or RhiM (0.4 μM) was added to a mixture containing 50 mM Tris-HCl pH 8.0, 100 mM NaCl, 12.5 mM substrate, 12.5 mM ATP and 12.5 mM MgCl_2_. The reaction mixture was incubated at room temperature overnight and then desalted using a ZipTip C18 (Merck Millipore). Equal volumes of the desalted sample and α-cyano-4-hydroxycinnamic acid (α-CHCA) were mixed on a plate and allowed to dry at room temperature. Mass spectra were obtained using AXIMA Performance (Shimadzu Corporation, Kyoto, Japan) with 100 laser shots at a laser power of 50 to 70, in reflection mode.

### Enzymatic assay

The amount of free inorganic phosphate produced by the enzymatic reaction was quantified based on a calibration curve of a phosphoric acid standard solution using the Malachite Green Phosphate Assay Kit (BioAssay Systems). A reaction mixture containing 50 mM Tris-HCl pH 8.0, 100 mM NaCl, 12.5 mM substrate, 12.5 mM MgCl_2_, and 0.65 mM ATP was prepared and sampled 30 min after initiation of the reaction by enzyme addition. The sample was diluted 50-fold with ultrapure water and mixed with the Working Reagent to stop the enzymatic reaction. The mixture was incubated at room temperature for 30 min, and then the absorbance at 620 nm was measured.

## Data availability

The coordinates and structure factors for ATP–RhiM, AMPPNP–Leu–RhiC, ADP–LeuPA–RhiC, and ADP–RhiC have been deposited in the Protein Data Bank, with accession numbers 26SM, 26SN, 26SO, and 26SP, respectively.

## Supporting information

This article includes supporting information.

## Conflict of interest

The authors declare that they have no conflicts of interest with the contents of this article.
