# Supplementary material for: Catalytic loop closure governs substrate selectivity in the rhizocticin biosynthetic ligases RhiM and RhiC
Source: J Biol Chem. 2026 Aug 12;302(10):113438. doi: 10.1016/j.jbc.2026.113438 (PMC13579937; doi:10.1016/j.jbc.2026.113438)
Supplement: Supplementary file 1 [file mmc1.docx]

**Supporting Information**

**Catalytic loop closure governs substrate selectivity in the rhizocticin biosynthetic ligases RhiM and RhiC**

*Mizuki Sakai^1, 2^, Ryosuke Masuda^3^, Yu Hirano^2, 4^, Taro Tamada^2, 4, 5, *^, Akira Nakamura^6, *^*

*^1^ Quantum Life Science Course, Graduate School of Science and Engineering, Chiba University, Inage, Chiba 263-8522, Japan.*

*^2^ Institute for Quantum Life Science, National Institutes for Quantum Science and Technology, Inage, Chiba 263-8555, Japan.*

*^3^ Department of Chemistry, Faculty of Science, Gakushuin University, Toshima, Tokyo 171-8588, Japan.*

*^4^ Center of Quantum Life Science for Structural Therapeutics, Chiba University, Inage, Chiba 263-8522, Japan.*

*^5^ Neutron Industrial Application Promotion Center, Comprehensive Research Organization for Science and Society, Tokai, Ibaraki 319-1106, Japan.*

*^6^ Department of Life Science, Faculty of Science, Gakushuin University, Toshima, Tokyo 171-8588, Japan*

* Corresponding authors

Taro Tamada: tamada.taro@qst.go.jp

Akira Nakamura: akira.nakamura@gakushuin.ac.jp

**Supporting Figures**

**Figure S1.** Overall structure of apo RhiM (PDB: 4WD3). The protomer adopts an ATP-grasp fold consisting of an N-terminal A-domain, an ATP-binding B-domain, and a C-terminal C-domain (subdivided into C1 and C2). Secondary structures are shown as cartoons.

**Figure S2.** Structure of the acyl phosphate intermediate and aminoacyl adenylate intermediate. (Left) Acyl phosphate intermediate in ATP-grasp amino acid ligases. The carboxylate group of the substrate amino acid attacks the γ-phosphate of ATP, forming an acyl phosphate intermediate upon the release of ADP. (Right) Aminoacyl adenylate intermediate in the adenylation domain of non-ribosomal peptide synthetases (NRPSs). The carboxylate group of the amino acid attacks the α-phosphate of ATP, generating an aminoacyl adenylate intermediate and releasing pyrophosphate (PPi). Although both enzymes activate the carboxylate group in an ATP-dependent manner, they form distinct chemical intermediates and release different leaving groups.

**Figure S3.** Gel filtration chromatograms. Chromatograms obtained using a Superdex 200 Increase 10/300 GL column. The elution volumes were 13.17 ml for RhiM and 13.65 ml for RhiC. Based on the amino acid sequence, the calculated molecular weight of the RhiM dimer is 94,800, and that of the RhiC dimer is 94,300, with each corresponding to its elution position. The expected elution volume for monomers of both RhiM and RhiC is approximately 15.7 ml. Elution positions of molecular weight markers are indicated with their molecular weight.

**Figure S4.** Superposition of apo and ATP-bound RhiM structures. The structures of apo-RhiM and ATP-bound RhiM are shown in gray and magenta, respectively. The bound ATP and magnesium ions are represented by stick and sphere models, respectively. The N-terminal and C-terminal ends and each domain are labeled.

**Figure S5.** Surface structures around the A-domain side of the active sites. The surface structures of RhiM (left) and RhiC (right) are colored by domain. ATP, AMPPNP, and l-Leu are shown as sticks. Mg^2+^ is represented by green spheres. SO_4_^2−^ in the crystallization conditions is shown as spheres. In RhiC, m*F*o−D*F*c electron density maps of SO_4_^2−^ contoured at 3.0σ is shown as a blue mesh. Lysine residues near the SO_4_^2−^ in RhiC and lysine residues that are structurally conserved in RhiM are shown as sticks.

**Figure S6.** Superposition of RhiC structures. (A) The structures of AMPPNP–Leu–RhiC, ADP–LeuPA–RhiC, and ADP–RhiC are shown in cyan, blue, and green, respectively. The bound AMPPNP, ADP, l-Leu, LeuPA, and Mg^2+^are represented by stick and sphere models, respectively. The N- and C-terminal ends, along with each domain, are labeled. (B) Close-up view of the active site. The color coding is the same as in (A).

**Figure S7.** MS/MS analysis of RhiC reaction products. MALDI-TOF MS/MS spectra in post-source decay mode for peptides generated by the RhiC reaction with l-Leu and l-Arg. The figure in the upper left shows the theoretical cleavage site of the precursor ion. Based on the assignment of fragment ions (indicated in stars), the precursor ions of *m*/*z* = 288.07, 401.11, and 514.16 were identified as L–R, L–L–R, and L–L–L–R, respectively.

**Figure S8.** MALDI-TOF MS analysis using the L–R dipeptide. The top row represents the reaction solution without the enzyme. The substrates used are (from the second row downwards): l-Leu + L–R, l-Val + L–R, and l-Val + l-Leu + l-Arg, respectively. The putative peptides generated and their m/z values are indicated in the figure.

**Figure** **S9.** Amino acid sequence alignment. Identical amino acids are highlighted with a black background, and similar amino acids are enclosed in a square. The numbers on the left indicate the residue positions. Secondary structures are shown above (RhiM) and below (RhiC). Helices are represented by cylinders and strands by arrows. The P-loop, ω-loop, and C-loop are labeled. The mutation sites are marked with stars.

**Figure S10.** Structural comparison of RhiM and RhiC. (A) Superimposition of the overall structures of ATP–RhiM (magenta) and AMPPNP–Leu–RhiC (cyan). (B) Close-up view of the active site. The color coding is the same as in (A).

**Figure S11.** Crystal structure of homologous l-amino acid ligase. Complex structure of BacD and an l-alanyl-l-phenylalanine analog (PDB ID: 3VMM). ADP and the analog are shown as sticks. Magnesium ions are represented as sphere models. Dashed lines indicate hydrogen bonds.

**Supporting Tables**

**Table S1.** Data collection and refinement statistics.

|  | **ATP–RhiM**^#^ | **AMPPNP–Leu–RhiC** | **ADP–LeuPA–RhiC** | **ADP–RhiC** |
| --- | --- | --- | --- | --- |
| **PDB ID** | 26SM | 26SN | 26SO | 26SP |
| **Data Collection** |  |  |  |  |
| Beamline | PF BL-17A | PF BL-1A | PF BL-1A | PF BL-5A |
| Wavelength (Å) | 0.98000 | 1.03500 | 1.02500 | 1.00000 |
| Space group | *C*2 | *P*4_2_2_1_2 | *P*4_2_2_1_2 | *P*12_1_1 |
| Cell dimensions |  |  |  |  |
| *a, b, c* (Å) | 108.9,  145.9,  84.1 | 191.2,  191.2,  132.6 | 192.2,  192.2,  134.7 | 131.2,  191.3,  191.3 |
| α, β, γ (°) | 90.0,  107.0,  90.0 | 90.0,  90.0,  90.0 | 90.0,  90.0,  90.0 | 90.0,  90.7,  90.0 |
| Resolution range (Å)^*^ | 44.07 – 2.45  (2.54 – 2.45) | 47.79 – 2.90  (2.99 – 2.90) | 48.04 – 3.04  (3.14 – 3.04) | 49.36 – 2.90  (2.95 – 2.90) |
| Total reflections^*^ | 600,071 (56,399) | 252,916 (22,515) | 656,352 (57,803) | 724,766 (34,906) |
| Unique reflections^*^ | 46,042 (4,497) | 52,355 (4,524) | 48,964 (4,423) | 208,523 (10,280) |
| Multiplicity^*^ | 13.0 (12.5) | 4.8 (5.0) | 13.4 (13.1) | 3.5 (3.4) |
| Completeness (%)^*^ | 100.0 (100.0) | 96.1 (96.9) | 99.9 (100.0) | 100.0 (100.0) |
| <*I*/σ(*I*)>^*^ | 13.1 (0.7) | 5.9 (1.1) | 6.5 (1.6) | 3.3 (1.2) |
| *CC*_(1/2)_^*^ | 0.999 (0.632) | 0.988 (0.558) | 0.971 (0.649) | 0.958 (0.629) |
| *R*_merge_ (%)^*^ | 13.6 (329.6) | 25.9 (237.5) | 34.8 (169.4) | 22.9 (100.9) |
| *R*_pim_ (%)^*^ | 3.9 (96.3) | 12.2 (111.5) | 9.8 (47.9) | 14.5 (64.4) |
| **Refinement** |  |  |  |  |
| Resolution (Å) | 44.07 – 2.45 | 47.79 – 2.90 | 48.04 – 3.04 | 49.36 – 2.90 |
| No. reflections  (work / free) | 43,773 / 2,228 | 49,743 / 2,576 | 46,439 / 2,495 | 197,760 / 10,728 |
| *R*_work_ / *R*_free_ (%) | 22.0 / 25.6 | 24.7 / 28.3 | 21.7 / 25.1 | 27.0 / 30.1 |
| No. atoms |  |  |  |  |
| Protein | 6,408 | 12,768 | 12,623 | 48,516 |
| Ligand, ion | 77 | 138 | 124 | 504 |
| Water | 22 | 10 | 18 | 301 |
| R.m.s. deviations |  |  |  |  |
| Bond lengths (Å) | 0.002 | 0.003 | 0.002 | 0.002 |
| Bond angles (°) | 0.486 | 0.587 | 0.520 | 0.607 |
| Ramachandran plot (%) |  |  |  |  |
| Favored | 95.8 | 96.8 | 96.1 | 95.1 |
| Allowed | 4.2 | 3.2 | 3.6 | 4.7 |
| Outliers | 0.0 | 0.0 | 0.3 | 0.2 |

*Values in parentheses correspond to the highest resolution shell.

\# Diffraction intensity data collected from two crystals were merged and scaled.

**Table S2.** Primers designed for cloning and mutagenesis.

|  | 5’-Primer-3’ |
| --- | --- |
| rhiC_NdeI_f | AGAAGGAGATATACATatgagcattttaatactgaacaaaacctc |
| pET-21a_XhoI_SLiCE_f | CTCGAGCACCACCACCACCACCACTGAGATCCGGC |
| rhiC_Y208F_f | aaattggccctctcggtTtataaacggatgtttgg |
| rhiC_Y208Q_f | aaattggccctctcggCaGataaacggatgtttgg |
| rhiC_E258A_f | caactacttttcacgcggCagtgtttcatactccg |
| rhiC_E258Q_f | caactacttttcacgcgCaagtgtttcatactccg |
| rhiC_E271K_f | gataagctagtgttttgtAagattgctagccggac |
| rhiC_R275A_f | gtgagattgctagcGCGactggaggggggt |
| rhiM_NdeI_f | AGAAGGAGATATACATatgcttcgtattttactcattaattccg |
| pET-21a_XhoI_SLiCE_f | CTCGAGCACCACCACCACCACCACTGAGATCCGGC |
| rhiM_R285A_f | cggtgagattgcaatgGCTattgggggcggaggg |
| rhiM_D376A_f | gcaaagttcaagttttGCTctggccaggctttatt |
| rhiM_D376L_f | gcaaagttcaagttttCTGctggccaggctttatt |
| rhiM_D376Y_f | gcaaagttcaagttttTATctggccaggctttatt |

Mutagenesis is indicated by capital letters. Only forward primers are listed, and the complementary strand is designated as the reverse primer.
